# Antimicrobial effectiveness of root canal sealers against *Enterococcus faecalis*

**DOI:** 10.1080/26415275.2022.2071719

**Published:** 2022-05-10

**Authors:** Paola Castillo-Villagomez, Elizabeth Madla-Cruz, Fanny Lopez-Martinez, Idalia Rodriguez-Delgado, Jorge Jaime Flores-Treviño, Guadalupe Ismael Malagon-Santiago, Myriam Angelica de La Garza-Ramos

**Affiliations:** Facultad de Odontología, Centro de Investigación y Desarrollo en Ciencias de la Salud (CIDICS), Universidad Autónoma de Nuevo León, Colonia Mitras Centro, Monterrey, Nuevo León, Mexico, CP, USA

**Keywords:** Root canal sealers, zinc oxide-eugenol, *Enterococcus faecalis*

## Abstract

We evaluated four root canal sealers to determine their antimicrobial effectiveness against *E. faecalis*. The direct contact test was used to measure the effectiveness of the study materials and close contact between bacteria on the kinetics of bacterial growth. The agar diffusion test (ADT) was also performed for comparison. Using one-way ANOVA and the F-test, significant differences between the sealers were confirmed. Whereas BioRoot endodontic sealer had an antimicrobial effect statistically similar to the zinc oxide-eugenol control (*p*=.99), EndoSequence sealer and AH Plus sealer both had a significantly lower antimicrobial effect than the control (*p*=.0000266 and *p*=.0000068, respectively).

## Introduction

Root canal treatment is based on debridement, disinfection, and filling of root canals [1]. Endodontics partially depends on the ability of the root canal sealers to prevent recurrent infection in apical periodontitis. Bacteria not only adhere to the walls of the main canals but also exist in branches, lateral canals, and dentinal tubules, where they are difficult to eliminate [2,3].

Complete removal of microorganisms from the root canal system in all patients is impossible; therefore, filling materials with antimicrobial activity for the root canal are used to reduce microorganisms and prevent infections. On the other hand, many endodontic failures occur after removing necrotic or inflammatory tissue with microorganisms. These tissues need to be retreated and managed with apical surgery; however, filtration failure occurs in 15% to 22% [4]. These complications are attributed to the lack of root canal sealing after endodontic treatment due to the high hydrophobicity and water absorption caused by the solubility of the cement. The development of new ceramic-type materials has improved sealing to reduce this problem. Epoxy resin is widely used as a gold standard, although it still has limitations, such as mutagenicity, cytotoxicity, inflammation, and hydrophobicity. Calcium silicate-based sealers with high biocompatibility and hydrophilicity have also been introduced. Both cements reduce microfiltration thanks to properties in their dynamic environment and being biocompatible in this application [5].

*Enterococcus faecalis* is a common microorganism found in persistent asymptomatic endodontic infections. Its prevalence in these infections is 24% to 77% [6]. Root canal treatment failure is usually caused by a refractory intracanal infection or microorganisms invading the canal from the apical portion of the tooth [6]. *E. faecalis* within the root canals is one of the organisms that may cause post-treatment failures [7]. This bacterial penetration is largely attributed to the limitations of current disinfection protocols to combat intracanal *E. faecalis* infection [8].

This study aimed to investigate the antibacterial effectiveness of the root canal sealers EndoSequence, BioRoot, AH Plus, and zinc oxide and eugenol (ZOE) against *E. faecalis* using the direct contact test (DCT). The hypothesis was that there would be no difference in antibacterial effectiveness between the four sealers.

## Methods

The sealers in this study were EndoSequence (calcium silicate-based sealer; Brasseler USA, Savannah, GA), BioRoot (calcium silicate-based sealer; Septodont, Saint-Maur-des-Fossés, France), AH Plus (epoxy resin-based sealer; Dentsply Sirona, Bensheim, Germany), and ZOE. The bacterial strain *E. faecalis* and root canal sealers ready for application were used. Samples with no *E. faecalis* growth, contaminated samples, and expired sealers were excluded. Samples that became contaminated during the procedure were removed. The antimicrobial effectiveness was evaluated using two tests. The agar diffusion test is commonly used to evaluate the antimicrobial activity of root canal sealers [9–11]. However, the agar diffusion test has been superseded by the DCT [12,13]. The DCT is a turbidimetric determination of bacterial growth kinetics that better reflects the antimicrobial potential of various sealers in standardized settings [14].

### Materials testing and microorganisms and their growth conditions

The root canal sealers were prepared according to the manufacturers' recommendations. Endosequence, Bioroot, and AH Plus were mixed and a coating was applied to the sidewall of the agar plates used for the experiments. *E. faecalis* ATCC 29212, which has been used to test the activity of antimicrobial endodontic materials, was used [14,15]. This isolate was obtained from the UANL Center for Research and Development in Health Sciences. Bacteria were grown aerobically from frozen stored cultures in brain heart infusion (BHI) broth at 37 °C. Laboratory prepared broths were used according to the manufacturer's specifications. Cells were harvested by centrifugation and resuspended in fresh medium. The inoculum was prepared by resuspending washed cells at predetermined optical densities related to known concentrations.

### Direct contact test

DCT was performed according to the Weiss et al. [16] method in 96-well microtiter plates. The study material (the sealers) was placed on the side wall of each well, taking care not to allow flow to the bottom of the well. Afterward, 10 µl of a bacterial suspension of 10^6^ cells was placed on the study material. This procedure guaranteed direct contact between the bacteria and the study material. Finally, BHI broth 245 µl was added to each well. Growth kinetics were evaluated with a microplate spectrophotometer at 595 nm at 37 °C in each well with recordings every 30 min. The negative controls were sealers with no bacterial inoculum. The positive controls were bacterial inoculum without sealers.

The negative control values were considered at baseline and were subtracted from the respective experimental sets. These were then plotted and statistically analyzed using one-way ANOVA and Scheffe's method of multi-point comparison.

### Agar diffusion test

A total of 200 µL of the bacterial suspension (approximately 10^6^ cells) was used for the agar diffusion test. The suspension was seeded on agar plates with BHI broth. Vertical wells 5 mm in diameter were drilled and filled with samples of each material. The plates were incubated at 37 °C for 24 h. After incubation, samples were examined for bacterial growth by measuring the inhibition halo on the agar. All experiments were done in triplicate. Repetitions were performed to test the experiment and the result of the set material after 24 h.

### Statistical analysis

One-way ANOVA and Scheffe's multi-point comparison were used for statistical analysis. The normal distribution of data was confirmed before one-way ANOVA. Dunnett's test was used to compare the sealers and the control. A *p*-value <.05 was considered significant.

## Results

### Direct contact test

BioRoot had a statistically similar antimicrobial effect to that of the ZOE positive control (*p*=.9999). EndoSequence and AH Plus sealer both had a significantly lower antimicrobial effect than the ZOE control (*p*=.0000266 and *p*=.0000068, respectively) (Table 1). The experiment was carried out with the bacteria (negative control) with a mean absorbance of 0.352 ± 0.0376 compared to ZOE (*p*=.9999373). The p-value of the negative control compared to Bioroot was .059863, with Endosequnce .066928, and with AH Plus .500167.

**Table 1.** Mean absorbance of study materials.

Substance	Mean	SD	SE	95% CI	Min	Max
**ZOE**	.03903	.000058	.000033	.03889–.03918	.039	.039
**AH Plus**	.85250	.198697	.140500	−.93272–2.63772	.712	.993
**EndoSequence**	.71750	.026163	.018500	.48244–.95256	.699	.736
**BioRoot**	.04467	.002887	.001667	.03750–.05184	.043	.048
**Bacteria**	.35233	.037554	.021682	.25904–.44562	.316	.391

SD: Standard deviation; SE, standard error; 95% CI: 95% confidence interval; Min, minimum; Max: maximum. ANOVA test results: *F* = 62.514; *p*-value .0000045.

Using Dunnet's test and treating ZOE as a control comparing it with the other groups, no significant difference was found between BioRoot and ZOE (*p*=.094), unlike the other treatments.

ZOE and bacteria without sealers were used as controls. A means analysis was also performed. It was possible to obtain the absorbance percentage (% Abs) of each sealer by removing 100% of the effect of the bacteria (the more positive the sealer, the greater the absorbance; on the other hand, the more negative the sealer, the lower the absorbance). ZOE had the highest negative absorbance. BioRoot was also negative. In contrast, AH PLUS had the highest positive absorbance followed by EndoSequence (Table 2).

**Table 2.** Direct contact test. Absorbance percentage according to the treatment substances.

Sealer	% mean vs. bacteria	% mean difference vs. bacteria	Mean absorbance vs. bacteria (*SD*)
ZOE (control)	11.08	−88.92	0.02
Bioroot	12.68	−87.32	0.82
Bacteria	100	–	10.66
Endosequence	203.64	103.64	7.43
AH Plus	241.96	141.96	56.39

Absorbance 595 nm.

### Agar diffusion test

BioRoot and EndoSquence had the largest zone of inhibition. BioRoot had a mean zone of inhibition of 4.333 ± 1.2 mm, EndoSequence 4.167 ± 1.17 mm, and AH Plus showed the smallest zone of inhibition with a mean of 3.167 ± 1.02 mm. Tukey's HSD test showed that there were no statistically significant differences between the sealers in the agar diffusion test.

## Discussion

In this study, we used EndoSequence (calcium silicate-based sealer), BioRoot (calcium silicate-based sealer), AH Plus (epoxy resin-based sealer), and ZOE to evaluate the ability of freshly mixed sealers to prevent the growth of microorganisms. These four sealers are the most commonly used root canal sealers. The antimicrobial activity of root canal sealers was evaluated against *E. faecalis* which is strongly associated with persistent periapical infection and endodontic failures [17]. It has been reported to be resistant to several antimicrobial agents [18] and it is a commonly used microorganism in *in vitro* studies. Orstavik [19] recommends the use of an endodontic sealer with antibacterial properties to decrease or avoid the future growth of microorganisms.

The DCT was performed based on the Weiss et al. [16] method, measuring the effect of close contact between the bacteria and the study material on growth kinetics using a microplate spectrophotometer. The agar diffusion test was performed for comparison. Weiss et al. [16] used two endodontic sealers, AH26 (epoxy resin type) and Endoflas (ZOE). Their results with the DCT were that Endoflas was a more potent bacterial growth inhibitor than AH26. In the agar diffusion test, AH26 produced a greater inhibition halo than Endoflas. In this study, BioRoot had a greater antibacterial effect.

Wang et al. [20] evaluated the antimicrobial effect of root canal sealers on *E. faecalis* biofilms in dentin tubules. We worked with *E. faecalis* in 96-well plates, not extracted teeth. Wang et al. [20] used three cements: AH Plus, EndoSequence, and the pulp canal sealer EWT (based on ZOE). The authors mention that the three endodontic sealers had antibacterial effects against *E. faecalis* in the dentinal tubules. The EndoSequence sealer and AH Plus had superior antibacterial effects compared to the EWT pulp canal sealer. In the present study, we found that EndoSequence and AH Plus had a similar antimicrobial effect against *E. faecalis*, but lower than BioRoot.

Singh et al. [21] also tested the antibacterial properties of root canal sealers against *E. faecalis*. They found that EndoSequence BC Sealer, MM mineral trioxide aggregate (MTA), and ProRoot MTA showed higher mean diameter inhibition halos. In contrast, MM-seal (epoxy resin-based) and Endoseal did not show inhibition. As in our study, the sealer with the lowest antimicrobial effect was the epoxy resin.

Zhang et al. [22] investigated the antibacterial activity of four root canal sealers against planktonic bacteria commonly detected in persistent and secondary endodontic infections. Zhang's study determined the antibacterial activity of the sealers AH Plus, TotalFill BC (bioceramic), RoekoSeal (silicone), and Guttaflow 2 (silicone) to detect biofilms cultured within 24 h. AH Plus had high antibacterial activity against all the investigated species, both in plankton and biofilms. In our study, we investigated only one bacterium. However, the antibacterial activity was lost after 24 h because AH Plus loses antibacterial activity after setting. [22] In our study, the root canal sealers BioRoot and EndoSquence (calcium silicate-based sealers) were more antimicrobial than AH Plus. This difference could be because we measured the antimicrobial effect in a 24-h period.

AlShwaimi et al. [23] conducted a systematic review. They summarized the results of *in vitro* studies of the antimicrobial effectiveness of root canal sealers against *E. faecalis* based on the DCT. Most of the studies reported that the different categories of freshly prepared sealers possessed positive antimicrobial activity against *E. faecalis* for up to 24 h, as in the present study. There was moderate evidence that no antimicrobial activity was found in aged sealer samples (2 to 7 days) in all categories. Evidence indicated positive antimicrobial activity of freshly mixed sealers against *E. faecalis*. Antimicrobial efficacy was lost as the material set. Unlike the Alshwaimi study, the present study did not analyze the antimicrobial effect after setting.

Based on the above-mentioned studies and the numerous others that have reported on similar experiments, we recommend that standardized *in vitro* methods should be developed to evaluate the antimicrobial activity of root canal sealers.

The four sealers in this study are commonly used. Knowing their antimicrobial activity can be of value in root canal treatment since they allow more functional dental rehabilitation.

## Data Availability

The datasets generated and/or analyzed during the current study are not publicly available due to ethical, legal, or commercial restrictions but are available from the corresponding author on reasonable request.
